# Loneliness and nervousness mediated the longitudinal association between sleep disorders and cyberbullying victimization in school-aged adolescents

**DOI:** 10.3389/fpsyt.2025.1640989

**Published:** 2025-11-24

**Authors:** Xin Xiao, Zu-Ling Jiang, Qiong Lei, Si-Jia Wang, Si-Xuan Li, Qing-Hai Gong

**Affiliations:** 1 Visual Science and Optometry Center of Guangxi, The People's Hospital of Guangxi Zhuang Autonomous Region, Nanning, Guangxi, China; 2 Scientific Research Department, The People's Hospital of Guangxi Zhuang Autonomous Region, Nanning, Guangxi, China; 3 Guangxi Key Laboratory of Eye Health, The People's Hospital of Guangxi Zhuang Autonomous Region, Nanning, Guangxi, China; 4 Guangxi Health Commission Key Laboratory of Ophthalmology and Related Systemic Diseases Artificial Intelligence Screening Technology, The People's Hospital of Guangxi Zhuang Autonomous Region, Nanning, Guangxi, China; 5 Scientific Research Department, The First Affiliated Hospital of Guangxi University of Chinese Medicine, Nanning, Guangxi, China; 6 Department of Ophthalmology, Nanxishan Hospital of Guangxi Zhuang Autonomous Region, Guilin, Guangxi, China; 7 Department of School Health, Ningbo Municipal Center for Disease Control and Prevention, Ningbo, Zhejiang, China

**Keywords:** sleep disorders, cyberbullying victimization, adolescents, loneliness, nervousness, mediation effect

## Abstract

**Background:**

This study investigated the longitudinal dose–response relationship between sleep disorders and cyberbullying victimization in school-aged adolescents and explored the mediating roles of psychological factors (loneliness, sadness, and nervousness).

**Methods:**

A 2-year longitudinal design was used to collect self-reported data on sleep disorders, physical activity, screen time, smoking, alcohol use, and dieting behavior. Cyberbullying victimization was assessed during follow-up. Multivariate logistic regression analysis and restricted cubic splines (RCSs) were used to analyze the dose–response relationship between sleep disorders and cyberbullying victimization. The mediation analysis explored indirect effects through loneliness, sadness, and nervousness.

**Results:**

Among the 1,910 adolescents (mean age: 12.2 ± 0.47 years), the mean sleep disorder score was 3.32 ± 3.68 (range: 0–27.0), and 196 (10.3%) engaged in cyberbullying victimization during the follow-up period. Sleep disorders were significantly associated with an increased risk of cyberbullying victimization (OR: 1.10, 95% CI: 1.06–1.14) after adjusting for confounders. Sensitivity analyses further validated the robustness of the results, which revealed that the risk of cyberbullying victimization increased approximately with increasing prevalence of sleep disorders. The RCS curve revealed that the risk of cyberbullying victimization increased approximately linearly with increasing prevalence of sleep disorders (P for overall<0.001, P for nonlinear=0.915). Compared with boys, girls with more sleep disorders presented a slightly greater risk of cyberbullying victimization (adjusted OR: 1.14 vs.1.08). Loneliness and nervousness partially mediated the association between sleep disorders and cyberbullying victimization, accounting for 25.00% (indirect effect β = 0.003, P < 0.001) and 8.33% (indirect effect β = 0.001, P = 0.038) of the total effect, whereas sadness had no significant effect.

**Conclusions:**

Sleep disorders independently predict cyberbullying victimization in adolescents, with stronger effects observed in girls. Loneliness and nervousness partially mediate this association. Targeted interventions to improve sleep, reduce loneliness and nervousness, and sex-specific strategies may mitigate cyberbullying victimization in school-aged adolescents.

## 1 Introduction

Sleep disorders among adolescents have emerged as a pressing global public health challenge, with meta-analyses estimating that approximately 34% (34%, 95% CI: 28–41%) of children and adolescents experience clinically significant sleep disturbances (1, 2). These deficits are associated with profound consequences, ranging from impaired neurocognitive development to heightened risks of mental health disorders (3–5). Concurrently, cyberbullying affects a substantial proportion of adolescents worldwide, with victimization rates reported ranging from 13.99% to 57.5%, and perpetration rates ranging from 6.0% to 46.3% (6–8). While previous studies suggest bidirectional links between sleep disruption and cyberbullying behaviors (9–11), the longitudinal mechanisms underlying this association remain poorly understood, particularly with respect to mediating psychological pathways and gender-specific vulnerabilities (9, 12).

Mounting evidence implicates sleep disorders as the precursors of cyberbullying (11). Neurobiological research has demonstrated that sleep deprivation disrupts prefrontal–amygdala connectivity—undermining emotion regulation and amplifying aggressive responses to social stressors (13–15). Conversely, cyberbullying victimization triggers hypervigilance and nocturnal rumination, thereby perpetuating sleep disturbances, with daily diary data illustrating sleep disturbance as a mediator between victimization and next-day negative affect (16, 17). However, three critical gaps limit current understanding (1): Most studies rely on cross-sectional designs, precluding causal inference (6, 10, 16) (2); the dose–response relationship between sleep disorder severity and cyberbullying risk remains unquantified; and (3) potential mediators—particularly distinct emotional states such as loneliness, sadness and nervousness—have not been systematically examined in longitudinal contexts (18, 19).

Psychological factors may mediate the link between sleep disorders and cyberbullying through various pathways. Loneliness, characterized by perceived social isolation, may exacerbate hostile attributions in peer interactions (20–22). Sadness—reflecting a dysphoric mood—can diminish motivation for prosocial conflict resolution (23), although older studies often conflated sadness with general emotional distress (24, 25). Nervousness (acute situational worry), although sometimes combined with anxiety, may operate differently, yet few recent studies have teased these aspects apart longitudinally. Our preliminary longitudinal data suggest that loneliness mediates 12.26% of the associations between sleep deficiency and fighting behavior (P < 0.05), whereas sadness and nervousness do not exhibit significant mediating effects (26). Notably, no longitudinal studies have compared these pathways while controlling for lifestyle confounders (e.g., screen time, substance use) that may covary with both sleep and cyberbullying (27).

This two-year prospective cohort study addresses these gaps through three objectives (1): to quantify the longitudinal dose–response relationship between baseline sleep disorders and incident cyberbullying victimization in Chinese adolescents (2); to test gender differences in this association, hypothesizing stronger effects in girls due to sex-specific emotional reactivity to sleep loss (28); and (3) to explore the influence of lifestyle factors (physical activity, screen time, smoking, alcohol use, and dieting behavior), as well as the mediating roles of loneliness, sadness, and nervousness. By employing restricted cubic splines (RCSs) for nonlinear exposure–outcome modelling and controlling for key behavioral confounders, this study advances the understanding of sleep disorder-cyberbullying mechanisms while informing targeted intervention strategies.

## 2 Methods

### 2.1 Study design and participants

**Figure 1:**
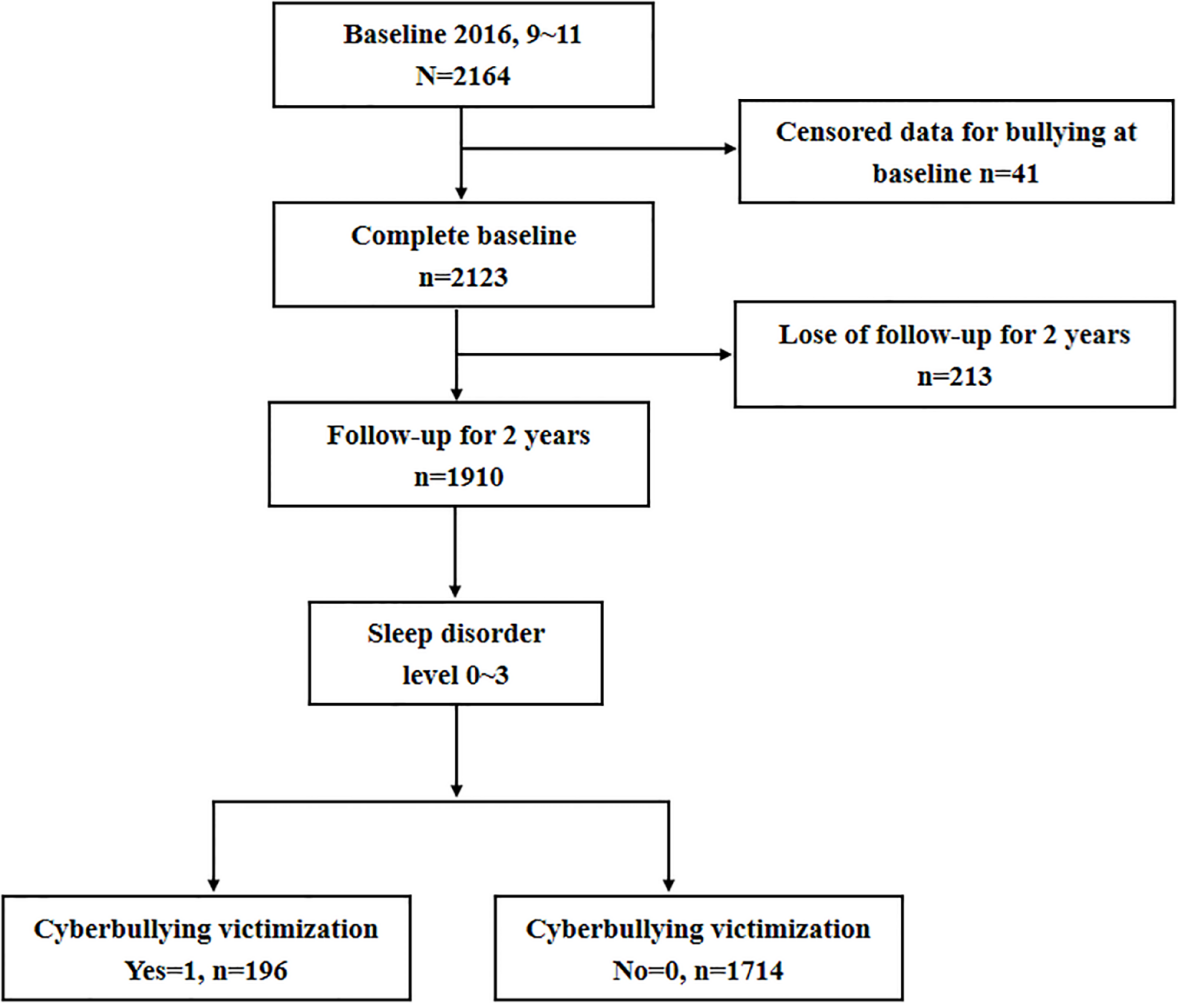
Flow chart of this study.

This school-based cohort study was conducted by the Ningbo Center for Disease Control and Prevention (CDC) in Ningbo, China. The baseline survey was administered beginning in October 2016 and included seventh-grade students from 13 middle schools across 10 districts via cluster random sampling. Follow-up assessments were conducted in October 2017 and October 2018. The study enrolled healthy adolescents and excluded those with major illnesses or incomplete data. The study process is illustrated in **Figure 1**.

### 2.2 Measures

A standardized, self-report questionnaire adapted from the U.S. Youth Risk Behavior Survey (YRBS) (29–31) was used to collect data on demographics, sleep duration and quality, physical activity, screen time, smoking, alcohol use, dieting behavior, and mental health. Trained investigators distributed and collected the questionnaires onsite, ensuring completeness.

#### 2.2.1 Sleep disorders

Sleep disorders were evaluated at baseline via the sleep disorders subscale of the Pittsburgh Sleep Quality Index (PSQI), developed by Buysee and colleagues (32), which is a self-administered questionnaire that measures sleep quality and disturbance over a 1-month period. There are nine items (5b to 5j) in this subscale dimension, ranging from 0 (none) to 3 (three or more times/week). The score of sleep disorders ranges from 0 to 27 points. The higher the score is, the greater the degree of sleep disorders. This sleep disorder evaluation method has been validated in a Chinese population (33). The Cronbach’s α for this subscale in the current sample was 0.717 (34).

#### 2.2.2 Cyberbullying victimization

Cyberbullying victimization was assessed in October 2017 and October 2018 by the following question: “During the past 12 months, have you been electronically bullied (e.g., via email, chat rooms, instant messaging, websites, or texting)?” (1=yes, 0=no) (18, 35). The Cronbach’s α for this subscale in the current sample was 0.738.

#### 2.2.3 Psychological factors

The psychological factors included self-reported loneliness, sadness, and nervousness (36). Psychological factors were assessed at Year 1 (2017). Loneliness was measured by the following question: “During the past 12 months, how often did you feel lonely?” with responses ranging from 0 (never) to 4 (always). Sadness was measured by the following question: “During the past 12 months, have you felt very sad or hopeless for two weeks or longer and stopped your daily activities?” (1 = yes, 0 = no) (37). Nervousness was measured by the following question: “During the past 12 months, have you felt very nervous, worried, or anxious for a week or longer?” (1 = yes, 0 = no) (37).

#### 2.2.4 Lifestyle factors and behavior

Lifestyle behaviors, including physical activity, screen time, smoking, alcohol use (38, 39), and dieting behavior, were evaluated at baseline. Physical activity was defined as engaging in moderate to vigorous exercise or muscle-strengthening activities at least once per week. Excessive screen time was defined as >2 hours per day of TV or internet use. Smoking and drinking were assessed on the basis of self-reported consumption at least once in the past month. Dietary behaviors included fruit and meat intake. Fruit intake was measured by the following question: “During the past 7 days, how many times did you eat fruits per day (excluding juice)?” The response options ranged from 1 (none) to 6 (5 or more times). Fruit intake was defined as engaging in at least once per day (1 = yes, 0 = no). A similar question was used to assess meat intake (39).

#### 2.2.5 Covariates

Demographic variables, including sex, age, parental marital status, and parental education level, were collected at baseline as potential confounders (40).

### 2.3 Statistical analysis

Descriptive statistics were computed for all the variables. Continuous variables were assessed for normality via the Kolmogorov–Smirnov test. Normally distributed variables are reported as the means ± standard deviations (SDs), whereas nonnormally distributed variables are expressed as medians and interquartile ranges (IQRs). Categorical data are presented as frequencies and percentages.

Between-group comparisons were performed via independent t tests or analysis of variance (ANOVA) for continuous variables and chi-square tests for categorical variables. Pearson correlation analysis or Spearman correlation analysis was used to explore the associations between sleep disorders and cyberbullying victimization, lifestyle factors, and psychological factors, and heatmaps were drawn.

Multivariate logistic regression models were applied to examine the association between sleep disorders and cyberbullying victimization, with the results reported as adjusted odds ratios (ORs) and 95% confidence intervals (CIs). To further explore the dose–response relationship between sleep disorders and cyberbullying victimization, we employed a restricted cubic spline (RCS) to further analyze their nonlinear relationship and plotted the RCS curve.

To assess the robustness of our results, we performed several additional sensitivity analyses. We classified sleep disorders into level 0 (sleep disorder score=0), level 1 (score=1–9), level 2 (score=10–18), and level 3 (score=19–27) groups on the basis of the sleep disorder score according to the PSQI instruction manual (32, 41) and calculated unadjusted and adjusted ORs and 95% confidence intervals (95% CIs) via univariate and multivariate logistic regression analyses.

Mediation analysis was conducted to explore the indirect effects of loneliness, sadness, and nervousness on the relationship between sleep disorders and cyberbullying victimization. All analyses were conducted via R software (version 4.5.1, R Foundation for Statistical Computing, Vienna, Austria). A two-tailed P value of <0.05 was considered statistically significant.

## 3 Results

### 3.1 Descriptive analysis

Among the 2164 students initially enrolled, 41 were excluded because of missing data, and 213 were lost to follow-up, resulting in a final sample of 1910 adolescents (**Table 1**). The mean age of the participants was 12.2 ± 0.47 years, and the mean sleep disorder score was 3.32 ± 3.68 (range: 0–27.0). Significant differences were observed among the sleep disorder groups in terms of sex, parental marriage status, TV screen time, internet screen time, alcohol use, fruit intake, and meat intake (all *P* < 0.05). Furthermore, adolescents with higher sleep disorder levels reported higher prevalences of loneliness, sadness, and nervousness (all *P* < 0.05).

**Table 1:** Baseline and follow-up characteristics by baseline sleep disorders (n=1910).

Characteristics	Level 0^*^ (score=0, n=529)	Level 1^*^ (score=1-9, n=1256)	Level 2^*^ (score=10-18, n=112)	Level 3^*^ (score=19-27, n=13)	*P* value
Age, years, mean (SD*)	12.2 (0.46)	12.2 (0.48)	12.2 (0.45)	12.3 (0.48)	0.515
Sex, n (%)					0.029
Girls	233 (44.0%)	641 (51.0%)	54 (48.2%)	4 (30.8%)	
Boy	296 (56.0%)	615 (49.0%)	58 (51.8%)	9 (69.2%)	
Parents’ marriage status, n (%)					0.025
Divorced/widowed/separated	43 (8.22%)	85 (6.87%)	10 (9.09%)	4 (30.8%)	
Married	480 (91.8%)	1153(93.1%)	100 (90.9%)	9 (69.2%)	
Parents’ education at college level, n (%)					0.452
Both had college degree	96 (19.5%)	148 (12.4%)	11 (10.5%)	1 (9.09%)	
Only one of them had college degree	70 (14.2%)	159 (13.3%)	15 (14.3%)	1 (9.09%)	
None of them had college	326 (66.3%)	886 (74.3%)	79 (75.2%)	9 (81.8%)	
Moderate physical activity (≥1 day/wk), n (%)					0.421
No	35 (6.62%)	95 (7.57%)	12 (10.7%)	1 (7.69%)	
Yes	494 (93.4%)	1160(92.4%)	100 (89.3%)	12 (92.3%)	
Mild physical activity (≥1 day/wk), n (%)					0.551
No	34 (6.43%)	76 (6.06%)	10 (9.01%)	0 (0.00%)	
Yes	495 (93.6%)	1179(93.9%)	101 (91.0%)	13 (100%)	
Muscle strengthening activity (≥1 day/wk), n (%)					0.097
No	184 (34.8%)	500 (39.8%)	51 (45.5%)	5 (38.5%)	
Yes	345 (65.2%)	755 (60.2%)	61 (54.5%)	8 (61.5%)	
TV screen (≥2 hour/day), n (%)					<0.001
No	427 (80.7%)	955 (76.0%)	74 (66.1%)	5 (38.5%)	
Yes	102 (19.3%)	301 (24.0%)	38 (33.9%)	8 (61.5%)	
Internet screen (≥2 hour/day), n (%)					<0.001
No	443 (83.7%)	1005(80.1%)	74 (66.1%)	8 (61.5%)	
Yes	86 (16.3%)	249 (19.9%)	38 (33.9%)	5 (38.5%)	
Cigarette use, n (%)					0.098
No	493 (93.2%)	1176(93.6%)	102 (91.1%)	10 (76.9%)	
Yes	36 (6.81%)	80 (6.37%)	10 (8.93%)	3 (23.1%)	
Alcohol use, n (%)					<0.001
No	347 (65.6%)	733 (58.4%)	46 (41.1%)	5 (38.5%)	
Yes	182 (34.4%)	523 (41.6%)	66 (58.9%)	8 (61.5%)	
Fruit intake (≥1 time/day), n (%)					<0.001
No	256 (48.4%)	752 (59.9%)	59 (52.7%)	6 (46.2%)	
Yes	273 (51.6%)	504 (40.1%)	53 (47.3%)	7 (53.8%)	
Meat intake (≥1 time/day), n (%)					<0.001
No	294 (56.0%)	824 (65.8%)	70 (62.5%)	5 (38.5%)	
Yes	231 (44.0%)	428 (34.2%)	42 (37.5%)	8 (61.5%)	
Loneliness, n (%)					<0.001
Never	318 (60.2%)	602 (47.9%)	29 (25.9%)	4 (30.8%)	
Occasion	150 (28.4%)	413 (32.9%)	43 (38.4%)	2 (15.4%)	
Sometime	31 (5.87%)	173 (13.8%)	22 (19.6%)	3 (23.1%)	
Regularly	15 (2.84%)	40 (3.18%)	13 (11.6%)	2 (15.4%)	
Always	14 (2.65%)	28 (2.23%)	5 (4.46%)	2 (15.4%)	
Sadness, n (%)					<0.001
No	472 (89.4%)	1059 (84.3%)	81 (72.3%)	9 (69.2%)	
Yes	56 (10.6%)	197 (15.7%)	31 (27.7%)	4 (30.8%)	
Nervousness, n (%)					<0.001
No	406 (76.9%)	867 (69.0%)	55 (49.1%)	5 (38.5%)	
Yes	122 (23.1%)	389 (31.0%)	57 (50.9%)	8 (61.5%)	
Cyberbullying victimization, n (%)					<0.001
No	489 (92.4%)	1129(89.9%)	90 (80.4%)	6 (46.2%)	
Yes	40 (7.56%)	127 (10.1%)	22 (19.6%)	7 (53.8%)	

^*^According to the PSQI instruction manual.

### 3.2 Incidence of cyberbullying victimization during follow-up

Over the two-year follow-up period, 196 adolescents (10.3%) engaged in cyberbullying victimization. The incidence of cyberbullying victimization was significantly associated with sex, parental marital status, muscle strengthening activity, alcohol use, meat intake, and adverse mental health outcomes, including loneliness, sadness, and nervousness (all *P* < 0.05, **Table 2**). Compared with girls (6.5%), boys were more likely to be involved in cyberbullying victimization (13.8%). Additionally, adolescents from households with divorced, widowed, or separated parents demonstrated an increased risk of cyberbullying victimization (19.0% vs. 9.5%). Compared with those not involved in cyberbullying victimization, adolescents involved in cyberbullying victimization presented significantly greater levels of loneliness, sadness, and nervousness (all *P* < 0.05).

**Table 2:** Characteristics of the study subjects according to cyberbullying status.

Characteristics	Total (n=1910)	No (n=1714)	Yes (n=196)	*P value*
Age, years, mean (SD*)	12.2 (0.47)	12.2 (0.46)	12.2 (0.54)	0.604
Sex, n (%)				<0.001
Girls	932 (48.8%)	871 (50.8%)	61 (31.1%)	
Boys	978 (51.2%)	843 (49.2%)	135 (68.9%)	
Parental marriage status, n (%)				0.001
Divorced/widowed/separated	142 (7.54%)	115 (6.80%)	27 (14.1%)	
Married	1742 (92.5%)	1577 (93.2%)	165 (85.9%)	
Parental education at college level, n (%)				0.268
Both had college degree	256 (14.2%)	233 (14.4%)	23 (12.4%)	
Only one of them had college degree	245 (13.6%)	213 (13.2%)	32 (17.3%)	
None of them had college	1300 (72.2%)	1170 (72.4%)	130 (70.3%)	
Moderate physical activity (≥1 day/wk), n (%)				0.815
No	143 (7.49%)	127 (7.41%)	16 (8.16%)	
Yes	1766 (92.5%)	1586 (92.6%)	180 (91.8%)	
Mild physical activity (≥1 day/wk), n (%)				1.000
No	120 (6.29%)	108 (6.30%)	12 (6.15%)	
Yes	1788 (93.7%)	1605 (93.7%)	183 (93.8%)	
Muscle strengthening activity (≥1 day/wk), n (%)				0.036
No	740 (38.8%)	650 (37.9%)	90 (45.9%)	
Yes	1169 (61.2%)	1063 (62.1%)	106 (54.1%)	
TV screen (≥2 hour/day), n (%)				0.134
No	1461 (76.5%)	1320 (77.0%)	141 (71.9%)	
Yes	449 (23.5%)	394 (23.0%)	55 (28.1%)	
Internet screen (≥2 hour/day), n (%)				0.207
No	1530 (80.2%)	1380 (80.6%)	150 (76.5%)	
Yes	378 (19.8%)	332 (19.4%)	46 (23.5%)	
Cigarette use, n (%)				0.060
No	1781 (93.2%)	1605 (93.6%)	176 (89.8%)	
Yes	129 (6.75%)	109 (6.36%)	20 (10.2%)	
Alcohol use, n (%)				<0.001
No	1131 (59.2%)	1041 (60.7%)	90 (45.9%)	
Yes	779 (40.8%)	673 (39.3%)	106 (54.1%)	
Fruit intake (≥1 time/day), n (%)				0.060
No	1073 (56.2%)	950 (55.4%)	123 (62.8%)	
Yes	837 (43.8%)	764 (44.6%)	73 (37.2%)	
Meat intake (≥1 time/day), n (%)				0.026
No	1193 (62.7%)	1086 (63.6%)	107 (55.2%)	
Yes	709 (37.3%)	622 (36.4%)	87 (44.8%)	
Loneliness, n (%)				<0.001
Never	953 (49.9%)	896 (52.3%)	57 (29.2%)	
Occasion	608 (31.8%)	542 (31.6%)	66 (33.8%)	
Sometime	229 (12.0%)	188 (11.0%)	41 (21.0%)	
Regularly	70 (3.67%)	55 (3.21%)	15 (7.69%)	
Always	49 (2.57%)	33 (1.93%)	16 (8.21%)	
Sadness, n (%)				<0.001
No	1621 (84.9%)	1478 (86.2%)	143 (73.3%)	
Yes	288 (15.1%)	236 (13.8%)	52 (26.7%)	
Nervousness, n (%)				<0.001
No	1333 (69.8%)	1234 (72.0%)	99 (50.8%)	
Yes	576 (30.2%)	480 (28.0%)	96 (49.2%)	

### 3.3 Correlations between cyberbullying victimization and covariates (sleep disorders, lifestyle and psychological factors)

**Figure 2:**
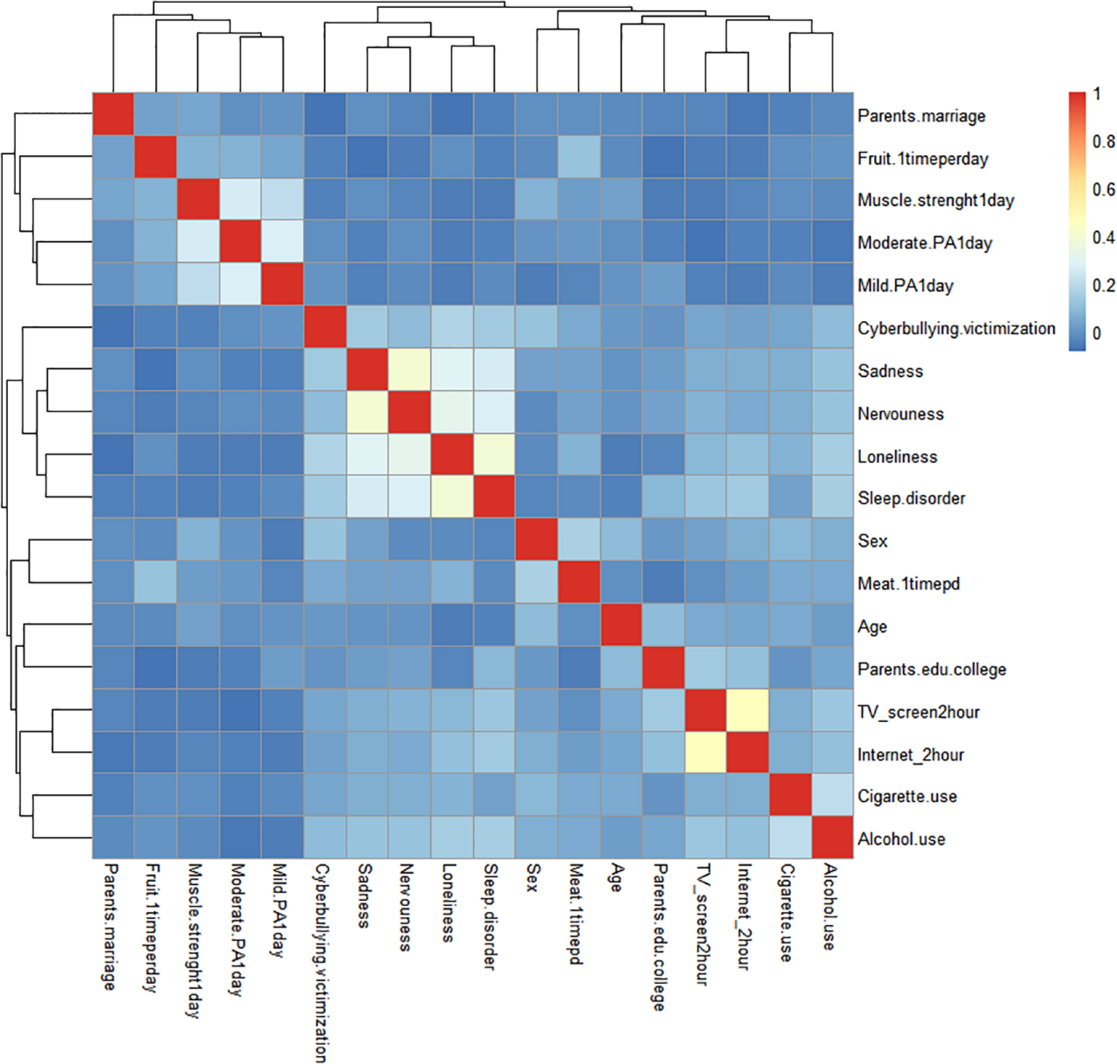
Heatmap of correlations between cyberbullying victimization and covariates (sleep disorders, lifestyle and psychological factors).

**Figure 2** shows the correlations of cyberbullying victimization with sleep disorders, lifestyle factors and psychological factors in school-aged adolescents. The heatmap correlation matrices revealed that cyberbullying victimization was significantly associated with sleep disorders, sex, parental marital status, TV screen time, internet screen time, alcohol use, fruit intake, meat intake, loneliness, sadness, and nervousness (all *P* < 0.05, **Table 2**, **Figure 2**).

### 3.4 Dose–response relationship between sleep disorders and cyberbullying victimization

**Figure 3:**
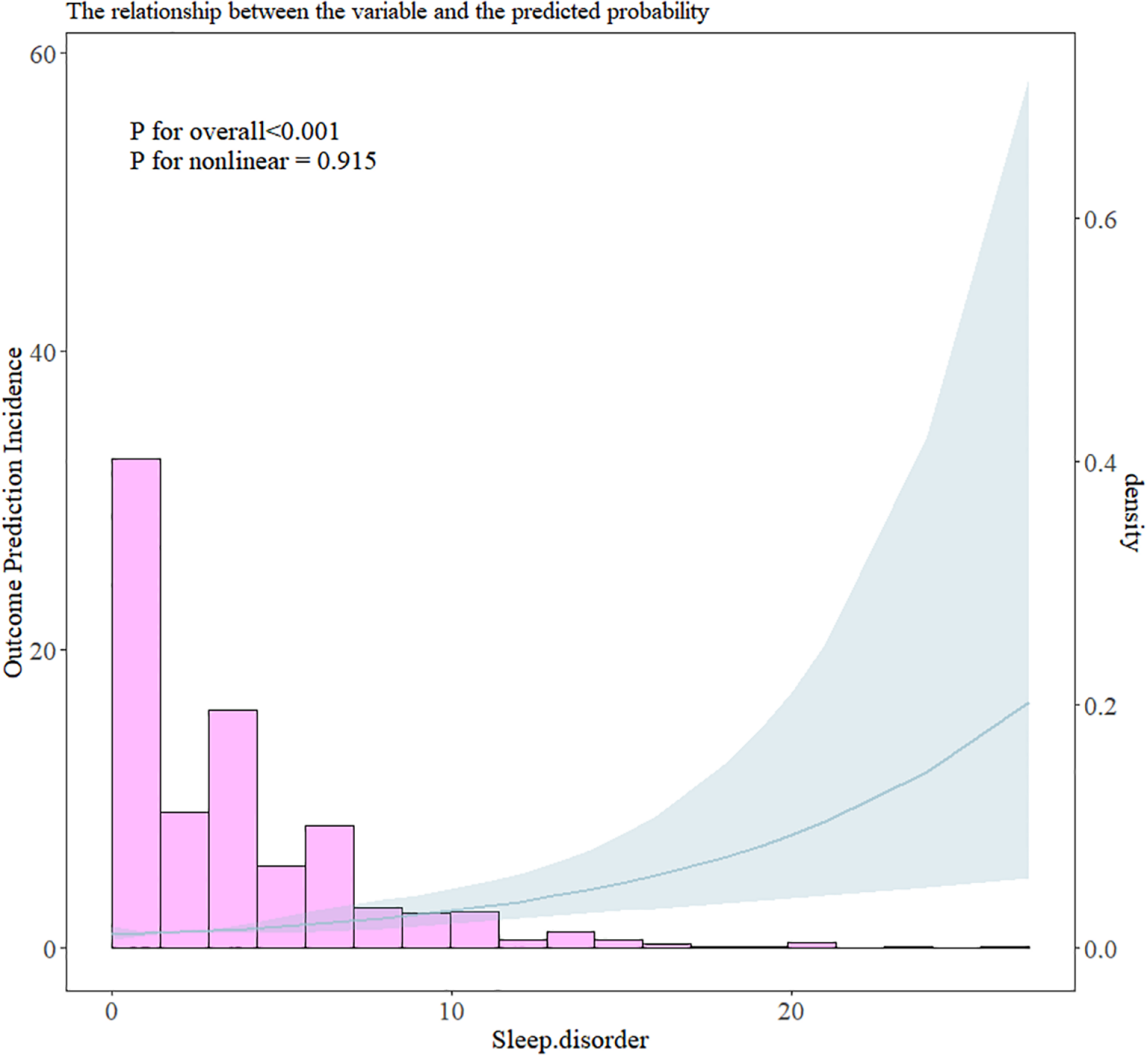
RCS plot of the relationship between sleep disorders and the risk of cyberbullying victimization.

Multivariate logistic regression demonstrated that adolescents with sleep disorders at baseline had a significantly greater risk of cyberbullying victimization during follow-up (OR: 1.11, CI: 1.07–1.14; **Table 3**). After adjusting for age, sex, parental marital status, education level, physical activity, screen time, cigarette and alcohol use, and dietary habits, sleep disorders remained an independent predictor of cyberbullying victimization (OR: 1.10, CI: 1.06–1.14). Sensitivity analyses further validated the robustness of the results, with the risk ratio of cyberbullying victimization increasing approximately with increasing incidence of sleep disorders (**Table 3**). The RCS curve revealed that the risk ratio of cyberbullying victimization increased approximately linearly with increasing incidence of sleep disorders (P for overall<0.001, P for nonlinear=0.915; **Figure 3**).

**Table 3:** Association between sleep disorders and cyberbullying victimization at follow-up.

Sleep disorders	Cyberbullying victimization		Crude		Multiadjusted	
	No	Yes	OR (95%CI)	*P value*	OR (95%CI)	*P value*
Total (n=1910)						
Sleep disorders			1.11(1.07-1.14)	<0.001	1.10(1.06-1.14)	<0.001
Sleep disorders level						
0	489	40	Ref.		Ref.	
1	1129	127	1.38 (0.96-2.01)	0.092	1.41 (0.95-2.14)	0.097
2	90	22	2.99 (1.67-5.23)	<0.001	2.55(1.33-4.76)	0.003
3	6	7	14.30(4.54-46.30)	<0.001	9.61(2.58-37.10)	<0.001
Boys (n=978)						
Sleep disorders			1.09(1.05-1.14)	<0.001	1.08(1.03-1.14)	<0.001
Sleep disorders level						
0	266	30	Ref.		Ref.	
1	529	86	1.44 (0.94-2.27)	0.104	1.42 (0.88-2.37)	0.163
2	43	15	3.09(1.51-6.16)	0.001	2.63(1.18-5.69)	0.015
3	5	4	7.09(1.68-28.20)	0.005	7.47(1.49-37.5)	0.012
Girls (n=932)						
Sleep disorders			1.15(1.08-1.21)	<0.001	1.14(1.07-1.22)	<0.001
Sleep disorders level						
0	223	10	Ref.		Ref.	
1	600	41	1.52(0.78-3.27)	0.244	1.36(0.68-2.99)	0.407
2	47	7	3.32(1.15-9.10)	0.020	2.83(0.87-8.57)	0.070
3	1	3	66.9 (7.82-1422.0)	<0.001	30.2 (1.99-813.0)	0.015

Adjustment for age, sex, parental marriage status, parental education at the college level, physical activity, screen time, cigarette use, alcohol use, and dieting (fruit intake, meat intake).

Gender-stratified analyses revealed that sleep disorders increased the risk of cyberbullying victimization among both boys (OR: 1.09, 95% CI: 1.05–1.14) and girls (OR: 1.15, 95% CI: 1.08–1.21). After adjusting for confounders, the association remained significant for both genders, although girls with greater sleep disorders presented a slightly greater risk of cyberbullying victimization (adjusted OR: 1.14, 95% CI: 1.07–1.22) than boys did (adjusted OR: 1.08, 95% CI: 1.03–1.14).

### 3.5 Mediation analysis

**Figure 4:**
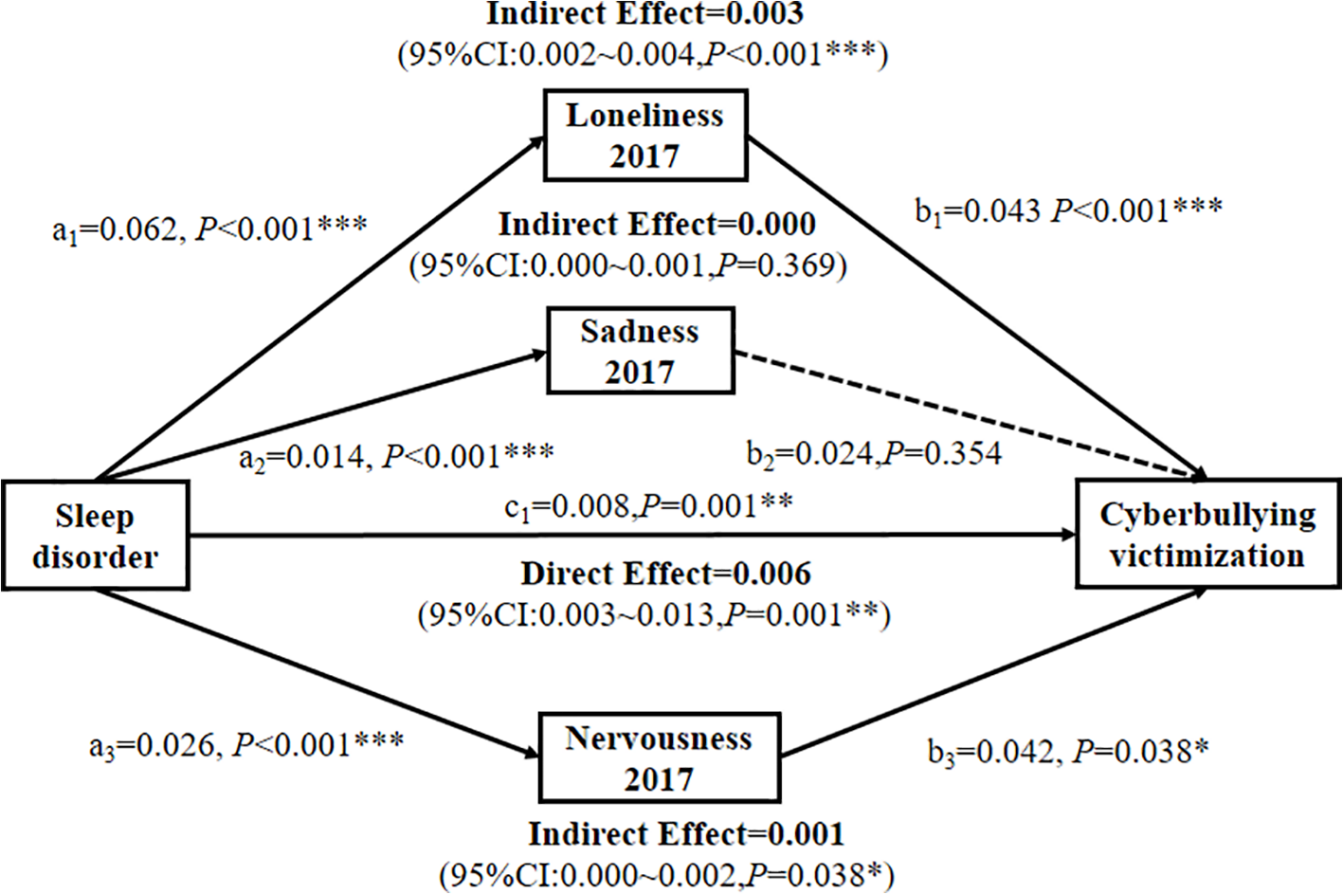
Multiple parallel longitudinal associations between sleep disorders and cyberbullying victimization are mediated by loneliness, sadness, and nervousness at Year 1 (2017). *p < 0.05; **p < 0.01; ***p < 0.001.

Mediation analysis was conducted to explore the roles of loneliness, sadness, and nervousness in the relationship between sleep disorders and cyberbullying victimization (**Table 4**, **Figure 4**). Sleep disorders were positively correlated with cyberbullying victimization (*β* = 0.008, *P* = 0.001) and significantly predicted higher levels of loneliness, sadness, and nervousness (all *P <* 0.05). Further analysis revealed that loneliness and nervousness were positively associated with cyberbullying victimization (*β* = 0.043 and 0.042, both *P <* 0.05).

**Table 4:** Mediation analysis of psychological factors (loneliness, sadness, and nervousness).

Type of effect	Mediation pathway	Effect size	SE	Bootstrap 95% CI	*P* value	Ratio of effect
Direct effect	Sleep disorders→cyberbullying victimization	0.008	0.002	0.003, 0.013	0.001	66.670%
Mediation Effect	Sleep disorders→loneliness→cyberbullying victimization	0.003	0.001	0.000, 0.002	<0.001	25.00%
	Sleep disorders→sadness→cyberbullying victimization	0.000	0.000	0.000, 0.001	0.369	0.00%
	Sleep disorders→nervousness→cyberbullying victimization	0.001	0.001	-0.000, 0.002	0.038	8.33%
Total Mediation Effect		0.004	0.001	0.003, 0.006	<0.001	33.33%
Total effect		0.012	0.002	0.007, 0.017	<0.001	

Loneliness partially mediated the relationship between sleep disorders and cyberbullying victimization, accounting for 25.00% of the total effect (indirect effect *β* = 0.003, *P* < 0.001; **Table 4**, **Figure 4**). Similarly, nervousness exhibited a partially mediated effect, contributing to 8.33% of the association (indirect effect *β* = 0.001, *P* = 0.038; **Table 4**, **Figure 4**). In contrast, sadness exhibited no significant mediating effects (*P* = 0.369), suggesting that the link between sleep disorders and cyberbullying victimization is driven primarily by increased loneliness and nervousness.

## 4 Discussion

### 4.1 Key findings

This two-year longitudinal study advances our understanding of the complex relationship between sleep disorders and cyberbullying victimization in school-aged adolescents. After adjusting for lifestyle and sociodemographic confounders, sleep disorders independently predicted cyberbullying victimization (adjusted OR: 1.10, 95% CI: 1.06–1.14), with a stronger association observed in girls (adjusted OR: 1.14 vs. 1.08 for boys). Notably, loneliness and nervousness mediated 25.00% and 8.33% of this association, respectively, whereas sadness had no significant mediating effect. These findings highlight the critical interplay between sleep disturbances, emotional states, and cyberbullying behaviors, emphasizing the need for integrated interventions targeting sleep health and psychological well-being to mitigate the risk of cyberbullying victimization.

### 4.2 Mechanisms linking sleep disorders to cyberbullying victimization

Our results align with theoretical frameworks suggesting that sleep disorders impair emotional regulation and executive functioning, thereby increasing vulnerability to aggression and peer conflict (42–44). The observed dose–response relationship (*P* for overall < 0.001, *P* for nonlinear = 0.915) supports prior evidence that even subclinical sleep disturbances increase the risk of cyberbullying (11, 45, 46). For example, experimental studies have demonstrated that sleep deprivation heightens amygdala reactivity to negative stimuli while reducing prefrontal cortex activity, fostering impulsivity and hostile social perceptions (47–49). Another cross-sectional survey by Chervin et al. (2003) demonstrated that adolescents with sleep-disordered breathing (SDB) exhibit 2–3 times more bullying and other specific aggressive behaviors (50–52), which is consistent with our observed OR of 1.10. The lower risk magnitude in our cohort may stem from methodological differences: while prior studies focused on sleep disturbances (assessed via the Adolescent Sleep-Wake Scale) (53), we measured broader sleep deficits (e.g., insomnia, sleep apnea and narcolepsy) via the PSQI, a validated tool for adolescent populations (Cronbach’s α = 0.717 in our sample) (54). These neurobiological changes may explain why adolescents with sleep disorders are more prone to misinterpret social cues, escalating conflicts into cyberbullying perpetration or victimization (9, 10).

The gender-specific risk pattern—higher vulnerability in girls—contrasts with cross-sectional studies reporting stronger sleep-aggression associations in boys (55, 56) but aligns with longitudinal data suggesting that girls’ emotional reactivity to sleep loss is amplified during adolescence (57). This disparity may reflect sex differences in stress hormone responses (e.g., cortisol dysregulation) (58) or sociocultural factors, such as girls’ greater reliance on peer relationships for emotional support (59, 60). Further research should disentangle biological (e.g., pubertal hormones) and environmental (e.g., gender norms) contributors to these differences.

### 4.3 Moderating role of lifestyle factors and behaviors

The relationship between sleep disorders and cyberbullying may be moderated by lifestyle factors, with different lifestyle behavior variables presenting varying risk levels among adolescents. Interestingly, alcohol use is a lifestyle factor associated with both increased sleep disturbance and increased cyberbullying risk: adolescents who consume alcohol are more likely to experience sleep disorders and cyberbullying victimization, reinforcing links between alcohol use, sleep disruption, and aggression (61). Surprisingly, reduced meat intake has been associated with increased sleep problems in recent dietary studies, yet in some contexts, it is correlated with lower engagement in aggressive behaviors, including cyberbullying—findings that suggest that dietary components may have complex, bidirectional associations with both sleep and peer conflict risk (62, 63). Moreover, while greater internet use time is consistently related to worsened sleep patterns (e.g., delayed sleep onset, lower sleep quality) (64), it does not always correlate with greater cyberbullying victimization in these same cohorts. A possible explanation is that these outcomes pertain to victimization rather than perpetration: high screen or internet time may increase exposure to online environments, but not all users become targets; moreover, lifestyle and dietary moderators may buffer or amplify risk differently for victims vs. perpetrators.

### 4.4 Mediating roles of loneliness and nervousness

The partial mediation by loneliness (25.00%) and nervousness (8.33%) highlights the importance of social-emotional pathways in the link between sleep disorders and cyberbullying. Sleep disturbances may intensify feelings of isolation by limiting opportunities for positive peer interactions, such as through daytime fatigue that hinders social engagement (44, 65). In contrast, nervousness may heighten negative appraisals of social situations, thereby increasing hostility (66, 67). These mechanisms align with the social information processing model, wherein sleep loss biases cognitive processing toward threat detection and retaliatory behaviors (68, 69).

The absence of mediation by sadness indicates that depressive symptoms may not directly influence cyberbullying victimization, contrasting with findings from cross-sectional studies (70, 71). This discrepancy could stem from differences in measurement (single-item vs. validated scales) or from the longitudinal design capturing the temporal precedence of mediators. Future studies should employ multidimensional mental health assessments to clarify these pathways. This distinction has practical implications: antibullying programs should prioritize social integration over worry reduction alone.

### 4.5 Implications for intervention

Our results advocate for multitiered interventions:

1. Primary prevention: School-based sleep hygiene programs (e.g., delayed school start times, reduced screen time before bed) could mitigate sleep disturbances and downstream cyberbullying risks (72).
2. Psychological Support: Cognitive–behavioral strategies targeting loneliness (e.g., social skills training) and sadness (e.g., emotion regulation techniques) may disrupt the mediation pathway (73).
3. Gender-Sensitive Approaches: Tailored interventions for girls—such as peer support groups addressing sleep-related emotional dysregulation—could address their heightened vulnerability.

### 4.6 Strength and limitations

The strengths of this study include its longitudinal design, adjustment for key confounders (e.g., substance use, screen time), and robust mediation analysis. However, several limitations must be acknowledged (1): Self-report bias and single-item assessment: Subjective measures and single-item assessments of sleep and cyberbullying may underreport sensitive behaviors. Objective tools (e.g., actigraphy, peer nominations) and repeat-item assessment can strengthen future work (74, 75) (2). Cultural specificity: The sample was drawn from a single Chinese region; replication in diverse populations is needed to assess generalizability (3). Temporal granularity: The 2-year follow-up may miss short-term fluctuations in mediators. Ecological momentary assessment can capture dynamic sleep–emotion–behavior interactions (40, 76).

### 4.7 Conclusion

Sleep disorders represent a modifiable risk factor for cyberbullying victimization in adolescents, with loneliness and nervousness serving as critical mediators. By integrating sleep promotion with psychosocial interventions targeting emotional well-being, policymakers and educators can disrupt the pathway from poor sleep to aggressive behaviors. Future research should test the efficacy of such strategies in randomized trials and explore the bidirectional relationships between sleep, emotions, and cyberbullying across developmental stages.

## Data Availability

Publicly available datasets were analyzed in this study. Upon reasonable request, the corresponding author will provide the dataset.
